# Exclusive breastfeeding and postpartum depression: A protective association that is not modified by feeding intentions

**DOI:** 10.1371/journal.pone.0340269

**Published:** 2026-01-06

**Authors:** Rachel A. Ryan, Lauren T. Berube, Andrea L. Deierlein

**Affiliations:** 1 Public Health Nutrition, School of Global Public Health, New York University, New York, New York, United States of America; 2 Department of Population Health at New York University Grossman School of Medicine, New York University Langone Health, New York, New York, United States of America; Universita Cattolica del Sacro Cuore Sede di Roma, ITALY

## Abstract

Approximately 13% of women in the United States (U.S.) experience postpartum depression (PPD), a mood disorder that negatively affects maternal and infant wellbeing. Breastfeeding may protect against PPD and breastfeeding intentions may moderate this relationship. This study examined the association between breastfeeding status and PPD symptoms and whether exclusive breastfeeding intentions moderate this relationship in low-income women in the U.S. We utilized data from the Special Supplemental Nutrition Program for Women, Infants, and Children (WIC) Infant and Toddler Feeding Practices Study-2, a longitudinal cohort study of caregiver feeding practices and nutrition outcomes among children enrolled in WIC. Exclusive breastfeeding intentions were measured prenatally; breastfeeding status at 1 month; and PPD symptoms at 3 months postpartum using the Edinburgh Postpartum Depression Scale (elevated PPD symptoms defined as EPDS ≥ 10). We conducted logistic regression to examine the association between breastfeeding status and elevated PPD symptoms. We stratified the analysis by exclusive breastfeeding intention-status groups to assess moderation. Adjusted models controlled for self-reported maternal ethnicity, annual household income, and smoking during pregnancy. The analytic sample included 2,022 mothers who identified their race as Black or African American (20.7%), White (58.8%), or other (20.6%), and their ethnicity as Hispanic (48.1%) or non-Hispanic (51.9%). The majority were ≥20 years old (88.2%), not married (68.2%), and had a high school education or less (62.7%). Compared to mothers who exclusively breastfed, those who combination fed (AOR, 2.03; 95% CI, 1.23–3.40) or exclusively formula fed (AOR 2.03; 95% CI 1.16–3.42) at 1 month had higher odds of elevated PPD symptoms. Exclusive breastfeeding intentions did not moderate this relationship. While exclusive breastfeeding was associated with a lower likelihood of experiencing elevated PPD symptoms, the majority of participants did not meet their exclusive breastfeeding intentions, highlighting the need for enhanced breastfeeding support.

## Introduction

Postpartum depression (PPD) is moderate to severe depression that occurs in the first year after birth, typically within the first 3 months [1]. Symptoms include persistent feelings of sadness, hopelessness, anxiety, or irritability and disturbances to sleeping and eating patterns [2]. The prevalence of PPD is estimated at 13% in the United States (U.S.), but tends to be higher among women who are younger (≤19 years old), belong to non-Hispanic racial minority groups, or participate in the Special Supplemental Nutrition Program for Women, Infants, and Children (WIC) during pregnancy [3]. PPD has been linked to suboptimal physical and psychological maternal health, developmental delays and overall health concerns in infants, and may negatively impact infant feeding practices and maternal-infant bonding [4].

While the exact cause of PPD is undetermined, the hormonal changes that occur after delivery and psychosocial factors, including prenatal depression and lack of social support, are important contributing factors [2]. Breastfeeding may help protect against PPD by regulating the dramatic hormonal shifts that occur early postpartum [5], through physiological mechanisms such as increased oxytocin levels, downregulation of the hypothalamic-pituitary-adrenal (HPA) axis, and decreased levels of estrogen and progesterone levels during lactation [5,6].

Two recent systematic reviews with meta-analyses, largely based on non-U.S. studies, reported lower risk of PPD among mothers who exclusively breastfed compared to those who partially or never breastfed (effect estimates ranging from 8% to 53% lower odds) [7,8]. A cross-sectional analysis of the 2016 Pregnancy Risk Assessment Monitoring System (PRAMS) found that any breastfeeding and breastfeeding duration were negatively associated with PPD risk among women in the U.S [9]. Additional research is needed to determine whether partial or exclusive breastfeeding drives this association.

Psychosocial factors, including breastfeeding experiences and emotions, may also play a role, either buffering or exacerbating maternal mood. For example, mothers who experience breastfeeding difficulties, such as pain or low milk supply, are more likely to report symptoms of postpartum anxiety or depression [10]. The role of a mother’s prenatal breastfeeding intentions and her ability to meet them should also be considered as unmet breastfeeding intentions have been linked to increased PPD risk [11]. In the U.S., there is significant pressure to breastfeed from society, healthcare professionals, and public health campaigns [12,13]. While most women in the U.S. initiate breastfeeding [14], many women experience problems or barriers to breastfeeding, and only approximately one third meet their intended breastfeeding duration [13,15]. Women who do not meet their breastfeeding intentions may experience feelings of guilt, shame, or failure, and be more prone to postpartum anxiety and depression [12,16]. Few studies have examined associations between breastfeeding intentions and PPD, generally finding that mothers with unmet breastfeeding intentions were more likely to experience symptoms of PPD (Edinburgh Postpartum Depression Scale scores of 10 or higher [11] or 13 or higher [17]), compared to those who met their intentions. However, study populations were outside the U.S. or lacked racial, ethnic, and socioeconomic diversity. In the current study, we use data from the WIC Infant and Toddler Feeding Practices Study-2 (ITFPS-2) to explore the association between early breastfeeding status and elevated PPD symptoms, and whether this relationship varies by exclusive breastfeeding intentions among a sample of low-income women in the U.S.

## Methods

The ITFPS-2 is a longitudinal cohort study conducted by the United States Department of Agriculture to assess caregiver feeding practices and nutrition outcomes among children enrolled in WIC [18]. The study received ethical approval from the institutional review boards at Westat and the University of California, Los Angeles [19]. Approval was also obtained from all relevant state health departments and local WIC agencies [19]. This analysis used de-identified data from the ITFPS-2, and no additional ethical approval was required. Detailed information on the study has previously been published [18]. Participant recruitment started on July 1, 2013 and ended November 18, 2013. A representative sample of WIC participants were enrolled in the study using a stratified two-stage sampling approach: first, 80 WIC sites were selected from 27 states and territories across the U.S.; second, participants were recruited in person from each WIC site [18].

Women were invited to participate if they met the following eligibility criteria: 1) English or Spanish speaking, 2) at least 16 years of age, and 3) enrolling in WIC for the first time during their current pregnancy or when their infant was less than 2.5 months old [18]. Screening, enrollment, and follow-up interviews were conducted in English or Spanish over the phone. A total of 4,367 women were screened, provided written informed consent, and enrolled in the study. For participants under the age of majority in their state, both parental or legal guardian consent and adolescent assent were obtained [19]. Follow-up telephone interviews were conducted prenatally and when the children were 1, 3, 5, 7, 9, 11, 13, 15, and 18 months and every 6 months after that until the child was 60 months old [18]. Baseline data collection occurred during the first follow-up interview (i.e., prenatal, 1-month, or 3-month, depending on when the participant enrolled) [18].

For the current analysis, we included participants who completed the prenatal, 1-month, and 3-month interviews, therefore, all baseline data was collected during the prenatal visit. There were 2,649 women who enrolled during pregnancy, of whom 2,082 completed all three interviews. We excluded women with missing data for exclusive breastfeeding intentions (n = 26), infant feeding at 1 month (n = 1), postpartum depression symptoms (n = 26), and covariates (n = 7). The final analytic sample was 2,022 women.

### Measurement

#### Exclusive breastfeeding intentions and breastfeeding status.

At the prenatal interview, participants respond to the following statement: “When my baby is one month old, I will be breastfeeding without using any formula or other milk.” Responses were measured on a 5-point Likert scale, ranging from “strongly agree” to “strongly disagree.” Women who selected “strongly agree” or “agree” were categorized as intending to exclusively breastfeed, while those who selected “neutral,” “disagree,” or “strongly disagree” were categorized as not intending to exclusively breastfeed.

At the 1 and 3-month interviews, participants were asked the following question: “Are you or anyone currently feeding the child breastmilk either from the breast or from a bottle, formula, or both?” Response options included “only breastmilk,” “both breastmilk and formula,” and “only formula.” Participants were categorized as exclusively breastfeeding, combination feeding, or exclusively formula feeding, respectively.

#### PPD symptoms.

The Edinburgh Postpartum Depression Scale (EPDS) [20], a validated and commonly used measure to screen for postpartum depressive symptoms [21], was administered during the 3-month interview. The EPDS includes 10 items that ask participants how they felt over the past 7 days. Each item was scored on a scale of 0–3 and summed for a total score (range: 0–30), with higher scores indicating more depressive symptoms. Total scores were dichotomized using a cut-point of 10, which indicates possible PPD [22] and has been used in previous literature [11,23]. Elevated PPD symptoms were defined as having an EPDS score of ≥10.

#### Covariates.

During the prenatal interview, mothers self-reported their maternal race as American Indian or Alaska Native, Asian, Black or African American, Native Hawaiian or Other Pacific Islander, White, or Other (write-in). For analysis, responses were categorized as Black, White or Other (which included American Indian or Alaska Native, Asian, Native Hawaiian or Other Pacific Islander, and write-in responses). Mothers also self-reported Hispanic ethnicity (yes or no); age (categorized as 16–19, 20–25, or ≥26 years), educational attainment (categorized as more than high school, yes or no), marital status (categorized as married, yes or no), birth country (U.S.; yes or no), annual household income (categorized as ≤75%, 76% to 130% and >130% of the 2013 poverty guidelines), smoking status during pregnancy (categorized as any smoking, yes or no), and number of live births (categorized as primiparous, yes or no). During the 1-month interview, mothers self-reported whether they had a single or multiple birth, their mode of delivery (vaginal or cesarean section), their infant’s birth weight (categorized as low: ≤ 5 lbs 9 oz; normal: >5 lbs 9 oz to <9 lbs 14 oz; or high: ≥9 lbs 14 oz), and preterm birth (infant born <37 weeks gestation, yes or no). During the 3-month interview, mothers self-reported whether they were currently working for pay full time (≥35 hours), part time, or not at all (categorized as employed or not employed). All covariates were categorized as defined before being released as part of the publicly available data set, except for employment status, smoking status during pregnancy, and number of live births, for which categorical groups were collapsed to create dichotomous variables.

### Statistical analyses

We used STATA release 18 (StataCorp LLC, College Station, TX) to conduct all data analyses [24]. We applied the 3-month cross-section survey weight and accompanying replicate weights using the *svy* command to account for the complex survey design [18]. We used Pearson’s chi-squared test of independence and calculated weighted percentages and 95% confidence intervals for maternal, pregnancy, and infant feeding characteristics by elevated PPD symptoms.

We conducted separate logistic regression models to examine the associations of exclusive breastfeeding intentions, breastfeeding status, and exclusive breastfeeding intention-status groups with elevated PPD symptoms. For the analyses, we stratified participants into the following exclusive breastfeeding intention-status groups: 1) intended to exclusively breastfeed, exclusively breastfed (reference group), 2) intended to exclusively breastfeed, did not exclusively breastfeed, 3) did not intend to exclusively breastfeed; exclusively breastfed, and 4) did not intend to exclusively breastfeed, did not exclusively breastfeed. We tested for interaction by including a term for exclusive breastfeeding intention and status. We identified potential covariates based on prior literature [25,26] and included them in the models if they were associated with either exclusive breastfeeding intentions or breastfeeding status and elevated PPD symptoms in bivariate analyses (p < 0.10). Multivariable models were adjusted for maternal self-reported ethnicity, marital status, annual household income, and smoking status during pregnancy. Maternal ethnicity was included to control for cultural beliefs and practices related to infant feeding [27,28]. We also conducted a sensitivity analysis using “did not intend and did not exclusively breastfeed” as the reference category. Statistical significance was set at *p* < 0.05.

## Results

Maternal, pregnancy, and infant feeding practice characteristics by exclusive breastfeeding intention-status groups are presented in Table 1. Participants identified their race as Black or African American (20.7%), White (58.8%), or other (20.6%), and their ethnicity as Hispanic (48.1%) or non-Hispanic (51.9%). The majority of participants were ≥20 years old (88.2%), were not married (68.2%), and had a high school degree or less (62.7%). Nearly a third of participants (31.4%) were born outside of the U.S. Almost half of participants (40.7%) were primiparous and a third had a cesarean section (33.1%). Eleven percent of infants were born preterm. At 1 month, 33.5% of infants were exclusively breastfed, 32.2% were combination fed, and 34.3% were exclusively formula fed. Twenty-eight percent of mothers intended to and exclusively breastfed, one third intended to but did not exclusively breastfeed, 6% did not intend to but exclusively breastfed, and 34% did not intend to and did not exclusively breastfeed at 1 month. Approximately 1 in 10 participants (9.5%) reported elevated PPD symptoms.

**Table 1:** Maternal and pregnancy characteristics and postpartum depression status by exclusive breastfeeding intention-status groups (unweighted *n* = 2,022; weighted n = 319,086).

	Total (unweighted *n* = 2,022)	Exclusive Breastfeeding Intention-Status Groups^h^			
		Intended-exclusively breastfed (unweighted *n* = 551)	Intended-did not exclusively breastfeed (unweighted *n* = 658)	Did not intend-exclusively breastfed (unweighted *n *= 115)	Did not intend-did not exclusively breastfeed (unweighted *n* = 698)
	**Unweighted *n* (Weighted %)**				
**Maternal characteristics**					
**Race** ^**a**^					
Black	451 (20.7)	93 (16.5)	132 (18.3)	29 (22.9)	197 (26.0)
White	1,235 (58.8)	367 (63.4)	381 (55.4)	65 (58.7)	422 (58.7)
Other	336 (20.6)	91 (20.1)	145 (26.3)	21 (15.3)	79 (15.3)
**Ethnicity**					
Hispanic	837 (48.1)	241 (50.6)	336 (56.9)	36 (35.5)	224 (39.8)
Non-Hispanic	1,185 (51.9)	310 (49.4)	322 (43.1)	79 (64.5)	474 (60.2)
**Married**					
Yes	607 (31.9)	225 (40.4)	195 (31.3)	38 (39.1)	149 (24.0)
No	1,415 (68.2)	326 (59.8)	463 (68.7)	77 (60.9)	549 (76.9)
**Age** ^**b**^					
16-19 years	228 (11.8)	47 (9.2)	76 (12.7)	15 (14.6)	90 (12.4)
20-25 years	831 (38.9)	239 (41.0)	252 (37.4)	49 (37.8)	291 (38.8)
≥26 years	963 (49.3)	265 (49.8)	330 (49.9)	51 (47.6)	317 (48.8)
**Household income** ^**c**^					
≤75% of poverty guideline	1,251 (62.1)	299 (55.0)	426 (64.5)	67 (59.1)	459 (66.3)
76% to 130% of poverty guideline	570 (27.3)	176 (30.2)	173 (26.3)	31 (25.3)	190 (26.3)
>130% of poverty guideline	201 (10.6)	76 (14.9)	59 (9.3)	17 (15.6)	49 (7.4)
**Educational attainment** ^**d**^					
High school or less	1,256 (62.7)	293 (55.3)	410 (63.3)	72 (63.9)	481 (68.1)
More than high school	766 (37.3)	258 (44.7)	248 (36.7	43 (36.1)	217 (32.0)
**Work status** ^**e**^					
Employed	634 (30.2)	161 (28.5)	198 (29.8)	37 (27.5)	238 (32.6)
Not employed	1,388 (69.8)	390 (71.6)	460 (70.2)	78 (72.5)	460 (67.4)
**U.S. Born**					
Yes	1,464 (68.6)	409 (70.2)	417 (60.0)	88 (73.0)	550 (75.0)
No	558 (31.4)	142 (29.8)	241 (40.0)	27 (27.1)	148 (25.0)
**Cigarette use during pregnancy**					
Yes	225 (9.01)	35 (5.4)	61 (7.1)	11 (8.0)	118 (14.2)
No	1,797 (90.9)	516 (94.6)	597 (92.9)	104 (92.0)	580 (85.8)
**Pregnancy characteristics**					
**Primiparous**					
Yes	836 (59.3)	322 (59.3)	375 (57.8)	57 (48.6)	432 (62.7)
No	1,186 (20.7)	229 (40.7)	283 (42.2)	58 (51.4)	266 (37.3)
**Singleton birth**					
Yes	1,987 (98.4)	547 (99.4)	642 (97.9)	113 (98.9)	685 (98.1)
No	35 (1.6)	4 (0.6)	16 (2.1)	2 (1.1)	13 (1.9)
**Mode of delivery**					
Vaginal	1,367 (66.9)	404 (73.6)	412 (62.9)	85 (71.0)	466 (64.7)
Cesarean	655 (33.1)	147 (26.4)	246 (37.1)	30 (29.1)	232 (35.3)
**Infant birth weight**					
Low	138 (6.8)	37 (5.9)	43 (6.7)	4 (4.7)	54 (8.1)
Normal	1,860 (92.0)	508 (92.7)	607 (92.1)	111 (95.3)	634 (90.8)
High	24 (1.1)	6 (1.4)	8 (1.2)	0 (0.0)	10 (1.1)
**Preterm** ^**f**^					
Yes	217 (11.0)	44 (7.5)	77 (12.0	11 (11.3)	85 (12.8)
No	1,805 (89.0)	507 (92.5)	581 (88.0)	104 (88.8	613 (87.2)
**Postpartum depression** ^**g**^					
Yes	206 (9.4)	36 (5.4)	70 (10.3)	10 (6.5)	90 (12.4)
No	1,816 (90.6)	515 (94.6)	588 (89.7)	105 (93.5)	608 (87.6)

^a^Other includes American Indian or Alaska Native, Asian, and Native Hawaiian or Other Pacific Islander.

^b^Maternal age when the infant was born.

^c^Assessed prenatally.

^d^Assessed prenatally.

^e^Assessed at 3 months.

^f^Born < 37 weeks gestation.

^g^Assessed using the Edinburgh Postpartum Depression Scale (EPDS). Elevated PPD symptoms is defined as EPDS ≥ 10.

^h^Exclusive breastfeeding intention was assessed prenatally with the statement: “When my baby is one month old, I will be breastfeeding without using any formula or other milk.” and Likert scale responses (1-strongly agree to 5-strongly disagree). Women who selected “strongly agree” or “agree” were categorized as intending to exclusively breastfeed. Breastfeeding status was assessed at 1 month.

Associations of exclusive breastfeeding intentions, breastfeeding status at 1 and 3 months, and intention-status categories with PPD symptoms are presented in Table 2. Results of the multivariable logistic regression analyses revealed that participants who combination fed (AOR, 2.03; 95% CI, 1.23–3.40) or exclusively formula fed (AOR, 2.03; 95% CI, 1.16–3.42) were more likely to report elevated PPD symptoms compared to those who exclusively breastfed at 1 month. A similar pattern was observed using breastfeeding status at 3 months, with both combination feeding (AOR, 2.21; 95% CI, 1.30–3.75) and exclusive formula feeding (AOR, 2.59; 95% CI, 1.52–4.41) associated with significantly higher odds of elevated PPD symptoms compared to exclusive breastfeeding. In the stratified analyses, participants who intended to exclusively breastfeed but did not (AOR, 1.98; 95% CI, 1.15–3.42), as well as mothers who did not intend to and did not exclusively breastfeed (AOR, 2.12; 95% CI, 1.24–3.61) were more likely to report elevated PPD symptoms compared to those who intended to and exclusively breastfed. No significant differences were observed between those who did not intend to but exclusively breastfed, and those who intended to and exclusively breastfed (AOR, 1.10 95% CI, 0.48–2.48). The interaction between exclusive breastfeeding intentions and exclusive breastfeeding status was not statistically significant (AOR, 0.94; 95% CI, 0.34–2.60). In a sensitivity analysis where mothers who did not intend to and did not exclusively breastfeed were the reference group, mothers who intended to and exclusively breastfeed were less likely to report elevated PPD symptoms (AOR, 0.47; 95% CI. 0.28–0.81). No significant associations were observed for the other groups.

**Table 2:** Associations of exclusive breastfeeding intentions, breastfeeding status, and intention-status groups with elevated PPD symptoms (unweighted *n *= 2,022; weighted *n* = 319,086).

	OR^d^ (95% CI)	AOR^e^ (95% CI)
**Exclusive breastfeeding intention** ^**a**^		
Intended	Ref	Ref
Did not intend	**1.48 (1.07–2.06)**	1.29 (0.94–1.76)
**Breastfeeding status** ^**b**^		
Exclusively breastfed (1 mo)	Ref	Ref
Combination fed (1 mo)	**1.91 (1.11–3.30)**	**2.03 (1.23–3.40)**
Exclusively formula fed (1 mo)	**2.42 (1.51–3.90)**	**2.03 (1.16–3.42)**
Exclusively breastfed (3 mo)	Ref	Ref
Combination fed (3 mo)	**2.16 (1.25–3.72)**	**2.21 (1.30–3.75)**
Exclusively formula fed (3 mo)	**3.01 (1.80–5.0)**	**2.59 (1.52–4.41)**
**Exclusive breastfeeding intention-status**		
Intended-exclusively breastfed	Ref	Ref
Intended-did not exclusively breastfeed	**2.03 (1.17–3.52)**	**1.98 (1.15–3.42)**
Did not intend-exclusively breastfed	1.23 (0.55–2.76)	1.10 (0.48–2.48)
Did not intend-did not exclusively breastfeed	**2.50 (1.50–4.18)**	**2.12 (1.24–3.61)**
**Exclusive breastfeeding intention-status** ^**c**^		
Did not intend-did not exclusively breastfeed	Ref	Ref
Intended-exclusively breastfed	**0.40 (.24–0.67)**	**0.47 (0.28–0.81)**
Intended-did not exclusively breastfeed	0.81 (0.54–1.22)	0.94 (0.63–1.39)
Did not intend-exclusively breastfed	0.49 (0.21–1.18)	0.54 (0.21–1.35)

^a^Intentions were assessed prenatally with the statement: “When my baby is one month old, I will be breastfeeding without using any formula or other milk.” and Likert scale responses (1-strongly agree to 5-strongly disagree). Women who selected “strongly agree” or “agree” were categorized as intending to exclusively breastfeed.

^b^Assessed at 1 month.

^c^Sensitivity analysis with “did not intend-did not exclusively breastfeed” as the reference group.

^d^OR = odds ratio.

^e^AOR = adjusted odds ratio; Models were adjusted for self-reported maternal ethnicity, marital status, poverty level, and smoking during pregnancy.

## Discussion

Our findings revealed that breastfeeding is associated with elevated PPD symptoms in a sample of low-income women in the U.S. Specifically, exclusive breastfeeding, but not partial breastfeeding, was associated with a lower likelihood of experiencing elevated PPD symptoms. Prenatal exclusive breastfeeding intentions did not modify this relationship.

This is the first U.S.-based study to compare exclusive breastfeeding, combination breastfeeding, and exclusive formula feeding in relation to PPD. Our findings suggest that the association between breastfeeding and elevated PPD symptoms was driven by exclusive breastfeeding. This aligns with previous systematic reviews and meta-analyses, which have reported stronger associations between exclusive breastfeeding and decreased risk of PPD compared to partial or no breastfeeding [7,8].

In this study, approximately a third of women who intended to exclusively breastfeed at 1 month did not meet this intention, which aligns with previous research among women in the U.S [13,15]. We stratified the analyses by exclusive breastfeeding intentions and breastfeeding status to assess whether exclusive breastfeeding intentions moderated the relationship between breastfeeding status and PPD symptoms. We observed higher odds of elevated PPD symptoms among women who did not exclusively breastfeed, regardless of whether they intended to, compared to those who intended to and exclusively breastfed. Breastfeeding behavior rather than intention or intention-behavior mismatch was the primary factor associated with elevated PPD symptoms in this sample. However, prior research has shown that breastfeeding difficulties and unmet goals can contribute to feelings of disappointment, guilt, and shame [12,16].

Some evidence indicates that how mothers respond to breastfeeding difficulties may influence their risk of PPD [29]. Mothers who approach breastfeeding challenges with self-compassion, which includes self-kindness rather than judgement, an understanding that they are not alone in their struggles, and finding a balance between suppressing emotions and allowing them to overpower them, may help protect against PPD [29]. In addition to general breastfeeding support, incorporating counseling techniques that promote self-compassion may help reduce the psychological burden of breastfeeding.

It’s also important to recognize that mothers who formula feed report experiencing guilt more frequently than those who breastfeed, particularly when they had intended to breastfeed during pregnancy [30]. Mothers who formula feed may also face stigma [31,32]. An experimental study revealed that mothers who formula fed were viewed less positively than those who breastfed, with stigma influenced by their stated intentions: those who planned to formula feed were evaluated more negatively [32]. However, a survey study found that mothers who planned to formula feed reported little personal or public stigma, while those who were unable to breastfeed and ultimately used formula experienced greater internalized stigma and felt perceived by others as failures [31]. Low-income mothers experiencing food insecurity may face added stress around infant feeding, particularly if they do not have enough breast milk and must rely on formula, which is costly and not always consistently available [33].

While our findings align with prior research on the association between breastfeeding status and PPD, they differ from studies that have linked unmet breastfeeding intentions to elevated PPD symptoms (EPDS score of 10 or higher [11] and 13 or higher [17]). Several factors may explain this discrepancy, including variations in how breastfeeding intentions, breastfeeding status, and PPD symptoms were measured, differences in the timing of assessment, and heterogeneity in study populations. Notably, breastfeeding intentions and status are highly correlated, making it essential to account for both when examining their relationship to PPD. A key strength of our study is the stratification by intention-status categories, which allowed us to disentangle the individual contributions of prenatal intentions and actual breastfeeding behaviors.

Strengths of this study include its longitudinal design, a large representative sample of WIC mothers, and the use of the EPDS to measure PPD symptoms. This study also has several limitations. We were unable to conduct sensitivity analyses using an alternate threshold for PPD symptoms (e.g., ≥ 13), as the publicly available data set provides only a dichotomous indicator based on the ≥ 10 cutoff. The group classified as “did not intend-exclusively breastfed” was relatively small (n = 115) and estimates for this group had wide confidence intervals that likely reflect limited statistical power. As for measurement limitations, our exposure did not differentiate between direct breastfeeding and feeding expressed milk, which may carry different physical and emotional demands, especially for low-income mothers with limited workplace flexibility or support. Work status was measured at 3 months postpartum, following the 1-month breastfeeding exposure. Because early feeding behaviors may influence employment decisions, the inclusion of this variable as a covariate should be interpreted with caution. In addition, we lacked data on important potential confounders such as prenatal depression and social support. Prior research indicates that prenatal depression is associated with lower breastfeeding initiation and shorter breastfeeding duration [5], making it difficult to assess the bidirectional relationship between breastfeeding and elevated PPD symptoms in this study. This dataset also did not include measures of intimate partner violence (IPV), which is relevant to both depressive symptoms and breastfeeding, so we were unable to account for IPV in these analyses. Other indicators of maternal wellbeing were also unavailable, limiting our ability to evaluate whether unmet breastfeeding intentions are associated with broader aspects of maternal health.

## Conclusions

Our study examined associations between early breastfeeding status and elevated PPD symptoms among low-income U.S. mothers, and whether these associations varied by exclusive breastfeeding intentions. We found that mothers who exclusively breastfed in the first months postpartum had lower odds of elevated PPD symptoms, while breastfeeding intentions did not significantly modify this relationship. Notably, a substantial proportion of mothers did not meet their exclusive breastfeeding intentions, emphasizing the importance of supporting mothers in achieving their feeding goals to promote maternal mental health.
